# Role of atrial high-rate episodes in stratifying thromboembolic risk: a multiple cut-off diagnostic meta-analysis

**DOI:** 10.3389/fcvm.2023.1289372

**Published:** 2023-11-07

**Authors:** Andrea Saglietto, Andrea Ballatore, Carola Griffith Brookles, Henri Xhakupi, Gaetano Maria De Ferrari, Matteo Anselmino

**Affiliations:** ^1^ Division of Cardiology, Cardiovascular and Thoracic Department, “Citta Della Salute e Della Scienza” Hospital, Turin, Italy; ^2^ Department of Medical Sciences, University of Turin, Turin, Italy; ^3^ Dipartimento di Medicina Interna, Università Degli Studi di Genova, Genova, Italia

**Keywords:** AHRE, atrial fibrillation, subclinical AF, cardiac implantable electronic device, thromboembolic risk

## Abstract

**Aims:**

Despite the high prevalence rate of atrial high-rate episodes (AHREs) detected using cardiac implantable electronic devices (CIEDs), clinical guidelines and consensus documents have disagreed on a universal AHRE definition and a temporal cut-off related to subsequent thromboembolic events. This diagnostic test accuracy meta-analysis aims to derive the optimal temporal threshold of clinically significant AHREs from the available literature.

**Methods:**

The PubMed/MEDLINE and EMBASE databases were screened for studies on CIED patients reporting the incidence of thromboembolic events related to at least one AHRE temporal cut-off. A total of 23 studies were included: 19 considering the longest single AHRE and four the AHRE burden, respectively. A random-effect diagnostic test accuracy meta-analysis with multiple cut-offs was performed. Two analyses were performed according to the AHRE temporal cut-off subtype (longest episode vs. cumulative burden).

**Results:**

The analysis on the longest single AHRE indicated 0.07 min as the optimal duration to differentiate AHRE associated or not with thromboembolic events [sensitivity 65.4% (95% CI 48.8%–79.0%), specificity 52.7% (95% CI 46.0%–59.4%), and area under the summary receiver operating characteristic curve (AUC-SROC): 0.62]. The analysis on AHRE burden indicated 1.4 min as the optimal cut-off [sensitivity 58.2% (95% CI 25.6%–85.0%), specificity 57.5% (95% CI 42.0%–71.7%), and AUC-SROC 0.60]. A sensitivity analysis excluding patients with a history of atrial fibrillation and including high-quality studies only yielded similar results.

**Conclusion:**

The presence of AHRE, rather than a specific duration, relates to an increased, albeit low, thromboembolic risk in CIED patients. Any AHRE should constitute an additional element in patient-specific thromboembolic risk assessment.

## Graphical Abstract

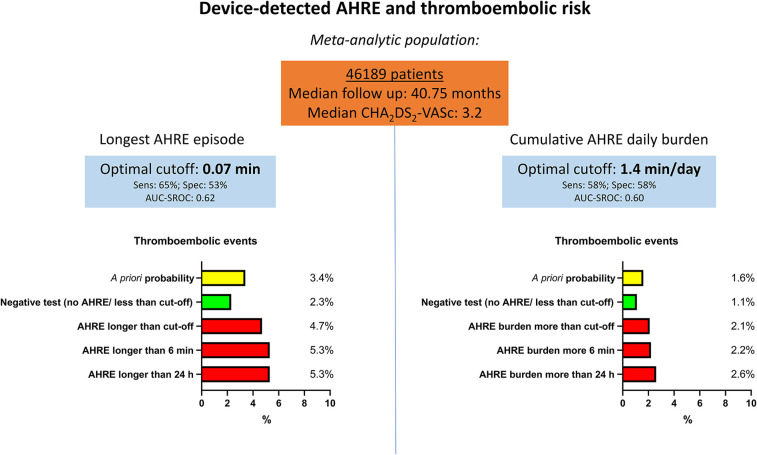

Graphical abstract reporting the main findings of the diagnostic meta-analysis.

## Introduction

Atrial high-rate episodes (AHREs) are frequently detected using cardiac implantable electronic devices (CIED) during routine clinical practice. Based on the population involved, the AHRE prevalence rate can range from 18% to 50% (1–3).

In recent years, several efforts were made to clarify the association of AHRE with an increase in thromboembolic risk and, subsequently, the potential need for anticoagulation (4). Despite clinical guidelines and consensus documents all agreeing on the importance of tracking these episodes, the proposed definitions are heterogeneous and have evolved over time: from episodes lasting more than 5–6 min of atrial beats above 180 beats per minute (bpm) (5) to any event, independent of duration, faster than 190 bpm (6), or at least 5 min at 175 bpm (7). Similarly, the recommendation for oral anticoagulation (OAC) in patients with AHRE has been advocated, but randomized evidence is lacking. AHRE burden, along with patient-specific risk factors, have been proposed as a guiding parameter in the choice of starting OAC based on the hypothesis that longer episodes would confer an increased risk. However, a clear AHRE duration cut-off has not been identified (4, 8, 9), neither a clinically significant threshold.

A diagnostic meta-analysis with multiple cut-offs is a recent development in the statistical field that has been successfully adopted in cardiovascular research (10, 11). The present study aims to perform a diagnostic meta-analysis to identify the AHRE temporal threshold most related to the thromboembolic risk of CIED patients.

## Methods

### Search strategy, study selection, and quality assessment

The PubMed/MEDLINE and EMBASE databases were screened from inception to November 2022, with the following search strategy: ((AHRE OR subclinical OR asympt* OR silent OR occult OR PM OR ICD OR implantable OR loop recorder OR continuous monitoring) AND (atrial fibrillation OR AF OR afib OR atrial tachyarrhythmia)) AND (stroke OR embo* OR thromboemb* OR TIA OR transient ischemic attack). The references of the relevant literature on the topic were also examined [including all the reports from the database search assessed for eligibility and the meta-analysis by Sagris et al. (12)]. Randomized controlled trials and prospective and retrospective studies were included if

• they reported the incidence of thromboembolic events [defined as stroke, transient ischemic attacks (TIA), and/or systemic thromboembolism] of patients with CIED (i.e., PM/ICD/CRT devices recording atrial activity) and
• they provided the sensitivity/specificity (or the associated raw counts) related to at least one cut-off time of AHRE associated with the incidence of thromboembolic events.

Studies were excluded if

• the implantable cardiac monitoring device was a loop recorder,
• more than 25% of the included patients presented clinical atrial fibrillation at baseline, and
• the investigated population involved cryptogenic stroke survivors.

Non-English language studies, abstracts, editorials, letters, systematic reviews, and unpublished data were excluded. Two investigators (AB and CG) independently reviewed the titles/abstracts and studies to determine their eligibility based on the inclusion criteria. They extracted all relevant features, including general characteristics, baseline population data, type of implanted CIED, follow-up strategy, AHRE definition (including time specification), and clinical outcomes. A third investigator (AS) solved any disagreements. Literature screening was performed with the help of a semi-automatic tool [Rayyan.ai (13); see Supplementary Material for details]. Quality assessment of the included studies was performed using the Newcastle–Ottawa scale: studies were deemed high-quality if the total score was ≥7/9.

### Primary outcome

The primary outcome of this diagnostic meta-analysis was defining the AHRE threshold most related to thromboembolic events. An ideal AHRE cut-off should theoretically differentiate between “aspecific” episodes and those significantly predicting clinical events. Thromboembolic events are defined as stroke, TIA, or systemic embolisms (SEs).

### Statistical analysis

The baseline characteristics of pooled study populations are reported as median values between the included studies, along with their interquartile range (IQR). The statistical instruments of a diagnostic test accuracy meta-analysis were adopted, treating the duration of AHRE as the “diagnostic test” (a positive test if the patients experienced an AHRE lasting at least as long as the cut-off time under examination) and evaluating the performance of this “diagnostic test” in predicting the “disease” (thromboembolic events) across multiple cut-offs. We extracted data from all included studies providing information on the AHRE duration in patients with CIED. We also constructed 2 × 2 tables for each identifiable cut-off time using the following definitions:

• true positives (TPs): patients with AHRE lasting at least as long as the cut-off time and suffering a thromboembolic event;
• true negatives (TNs): patients without AHRE or with AHRE lasting less than the cut-off time and not suffering a thromboembolic event;
• false positives (FPs): patients with AHRE lasting at least as long as the cut-off time and not suffering a thromboembolic event;
• false negatives (FNs): patients without AHRE or with AHRE lasting less than the cut-off time and suffering a thromboembolic event.

Supplementary Material provides a detailed description of how the data were extracted for each study. Two analyses following the AHRE definition (single longest episode vs. cumulative burden during a 24 h monitoring period) and sensitivity analyses including patients without previous atrial fibrillation (AF) and high-quality studies were performed. The R package *diagmeta* (14), which implements the method suggested by Steinhauser et al. (11) for the meta-analysis of diagnostic test accuracy studies with multiple cut-offs, was used to perform a multi-level random-effect diagnostic meta-analysis. Briefly, this meta-analytic approach is based on the estimation of the distribution functions of the underlying “marker” (in the present case, AHRE duration) within the non-diseased (patients not suffering from thromboembolic events) and diseased (patients suffering from thromboembolic events) individuals. Assuming a logistic distribution, the condition is estimated in both groups, applying a linear mixed effects model to the transformed data, accounting for across-study heterogeneity and dependence on sensitivity and specificity. The final output of the model is a summary receiver operating characteristic (SROC) curve. The resulting sensitivity and specificity at every potential threshold and the area under the SROC curve (AUC-SROC) are then computed. This approach also enables the determination of an optimal threshold across studies by maximizing the Youden index. Finally, to estimate the positive (PPV) and negative (NPV) predictive values of the computed optimal meta-analytic threshold, the prevalence of the “disease” (the prevalence of thromboembolic events in the entire CIED population) was obtained by pooling the study-specific prevalence using a random-effect model (*metaprop* function of R package *meta*).

All analyses were performed using R software version 4.2.2 (R Foundation for Statistical Computing, Vienna, Austria). A *P*-value of 0.05 was considered statistically significant.

## Results

**Figure 1:**
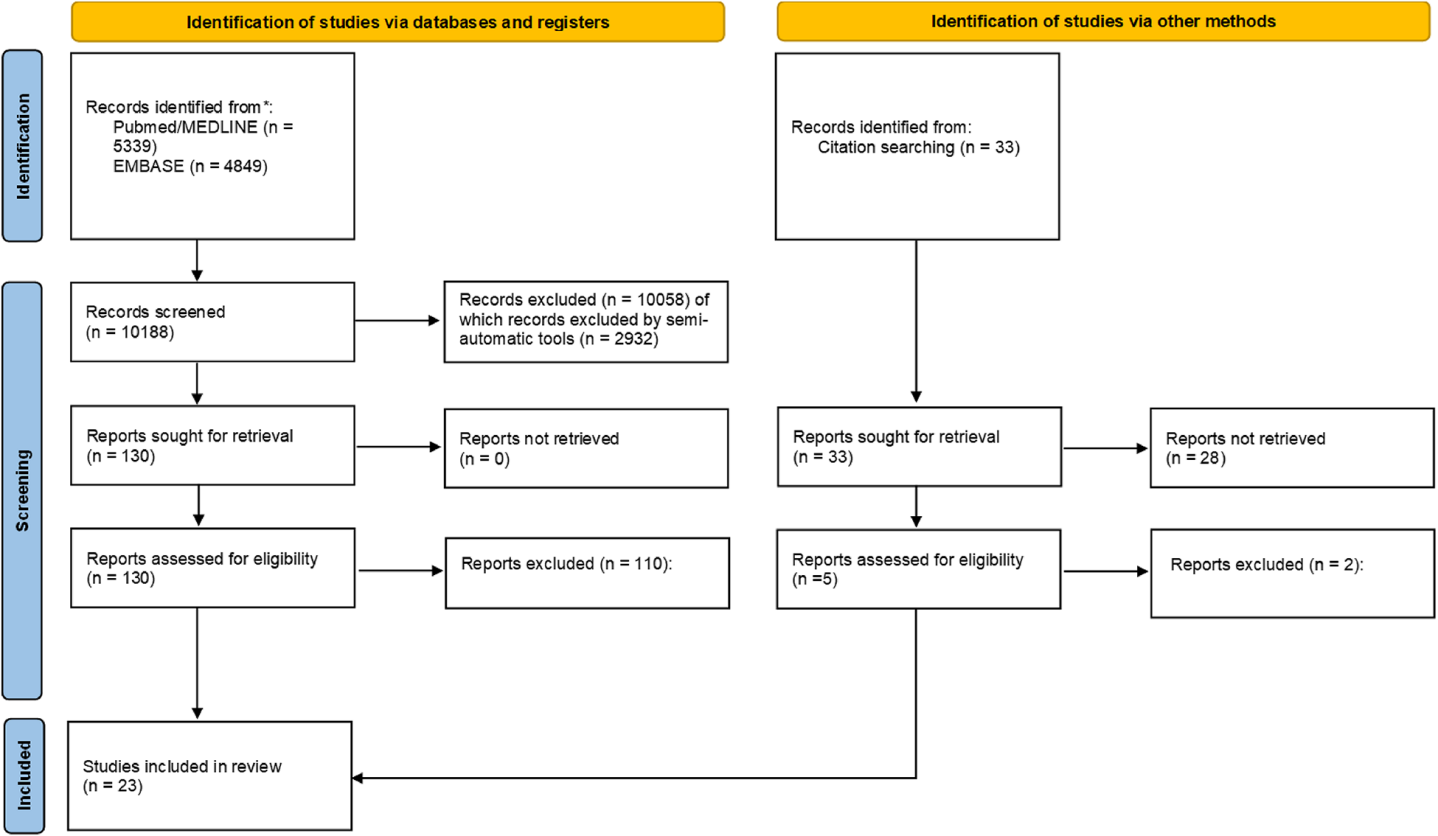
PRISMA flow chart.

The search retrieved 10,188 results; 135 articles were assessed for eligibility, and 23 (2, 15–36) were included in the final analysis. Figure 1 reports the PRISMA flow chart. The Supplementary Material describes the selection process, including the bibliographic references of the included studies. Supplementary Table S1 reports the study quality assessment using the Newcastle–Ottawa scale. Supplementary Table S2 reports the study-specific procedural details (e.g., type of CIED), follow-up strategies, and AHRE definitions.

Out of 46,189 patients with CIED, 1,167 experienced a thromboembolic event (3.0%, 95% CI 2.2–4.0). The median follow-up was 40.75 (IQR 28.25–52.80) months, with a minimum duration of 12 months. Table 1 reports the baseline characteristics. The median age was 70.0 years (IQR 66.6–75.5), with nearly two-thirds of male patients (males 60.6%, IQR 53.0%–69.0%). Hypertension (57.7%, IQR 45.3%–72.2%) is the most frequent concurrent comorbid condition, whereas heart failure (15.4%, IQR 5.9%–38.5%; median left ventricular ejection fraction of the entire population 54.0%, IQR 29.0%–65.0%) and coronary/ischemic heart disease (25.0%, IQR 17.5%–34.2%) were present in a minority of patients. The median left atrial antero-posterior diameter was 39.8 mm (IQR 38.0–41.0 mm). A median of 8.7% (IQR 6.9%–12.1%) of patients had a history of previous stroke/TIA or systemic thromboembolism. The median CHA_2_DS_2_-VASc score was 3.2 (IQR 2.8–3.8); 3.1% (IQR 0–10.2) were on oral anticoagulants at baseline.

**Table 1:** Baseline characteristics.

Variable	Median (IQR)
Age (years)	70 (66.6–75.5)
Male gender (%)	60.6 (53.0–69.0)
Hypertension (%)	57.7 (45.3–72.2)
Diabetes (%)	25.0 (21.0–29.4)
Dyslipidemia (%)	53.8 (40.2–73.0)
Coronary/ischemic disease (%)	25.0 (17.5–34.2)
Vascular disease (%)	12.5 (7.0–24.6)
Valvular disease (%)	31.2 (20.6–41.8)
CHA_2_DS_2_-VASc score	3.2 (2.8–3.8)
CHADS score	2.2 (1.9–2.3)
Beta-blocker use (%)	34.9 (24.8–68.6)
Baseline OAC (%)	3.1 (0.0–10.2)
History of AF (%)^a^	0.0 (0.0–0.0)
Echocardiographic parameters	
EF (%)	54 (29.0–65.0)
LA diameter (mm)	39.8 (38.0–41.0)

^a^ Mean value 4.1% (minimum–maximum interval: 0.0%–24%).

Supplementary Tables S3, S4 report TP, TN, FP, and FN counts in the included studies at each evaluated cut-off for single longest AHRE and cumulative AHRE burden analyses, respectively. Supplementary Tables S5, S6 report the distribution of temporal cut-offs for the primary analyses. The pooled prevalence rate of thromboembolic events in patients with CIED recording single episodes and AHRE burden was 3.4% (95% CI 2.5%–4.6%) and 1.6% (95% CI 1.0%–2.4%), respectively. In five out of 19 studies, 2 × 2 tables were designed for at least two cut-off times, with the study by Nakano et al. (27) providing the highest number of cut-offs (7). A meta-analysis of diagnostic test accuracy studies with multiple cut-offs for the analysis of the single longest AHRE [19 studies: total number of cut-offs 36, number of different cut-offs 11, and median follow-up 49.1 months (IQR 37.5–57)] indicated 0.07 min as the optimal duration to differentiate AHRE associated or not to thromboembolic events. The sensitivity and specificity at optimal cut-offs were 65.4% (95% CI 48.8%–79.0%) and 52.7% (95% CI 46.0%–59.4%), respectively, with an AUC-SROC of 0.62. The analysis on cumulative AHRE burden [four studies: total number of cut-offs 9, number of different cut-offs 8, and median follow-up 17.9 months (IQR 12.6–23.3)] indicated 1.4 min as the optimal burden to differentiate AHRE associated or not to thromboembolic events. The sensitivity and specificity at optimal cut-offs were 58.2% (95% CI 25.6%–85.0%) and 57.5% (95% CI 42.0%–71.7%), respectively, with an AUC-SROC of 0.60.

**Figure 2:**
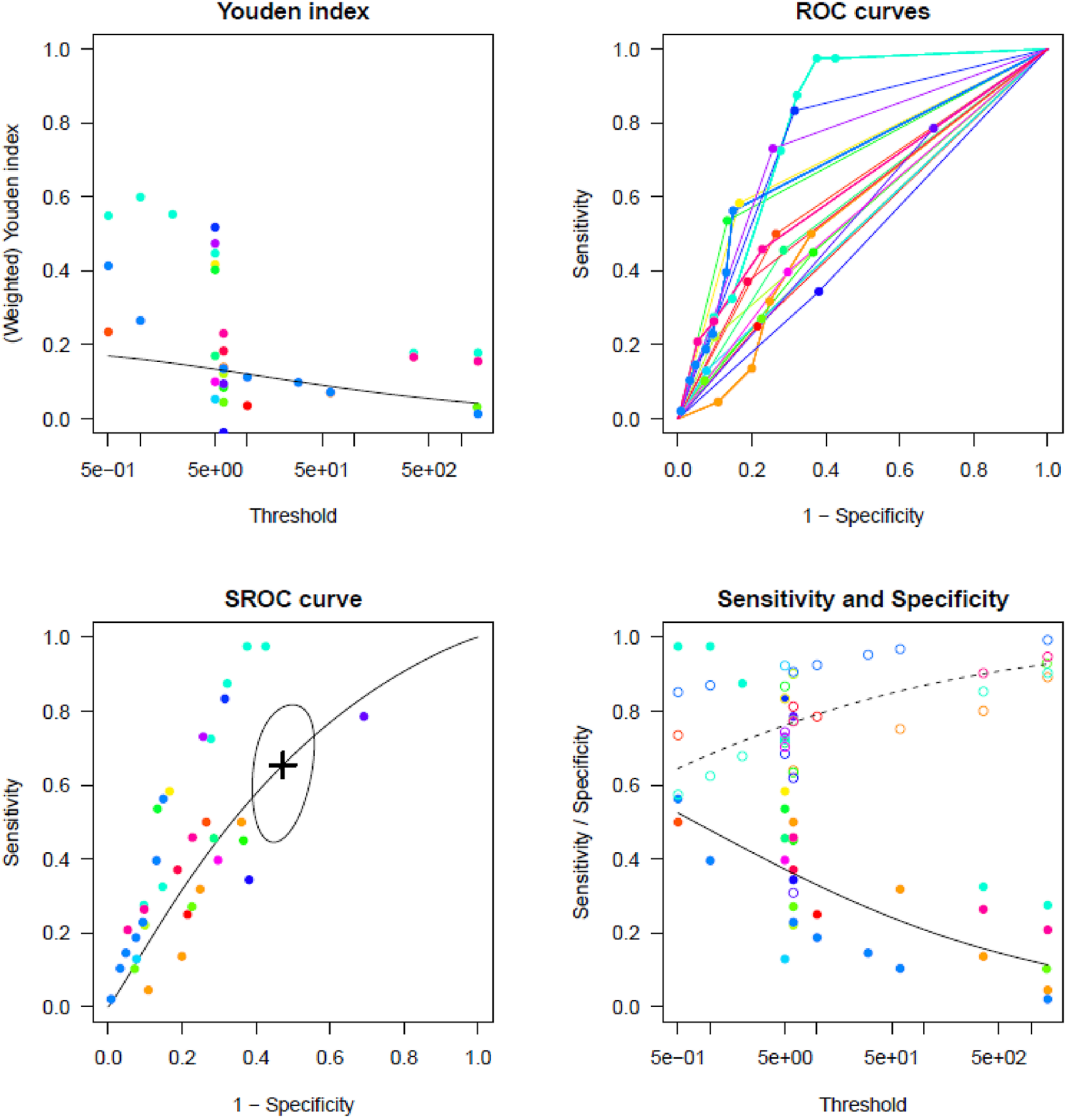
Meta-analysis of diagnostic test accuracy studies with multiple duration cut-offs assessing the differential performances of various single longest AHRE thresholds in predicting thromboembolic events. (**A**) Youden index as a function of test thresholds; (**B**) study-specific ROC curves; (**C**) meta-analytic SROC curve; (**D**) meta-analytic sensitivity/specificity as a function of different test thresholds.

**Figure 3:**
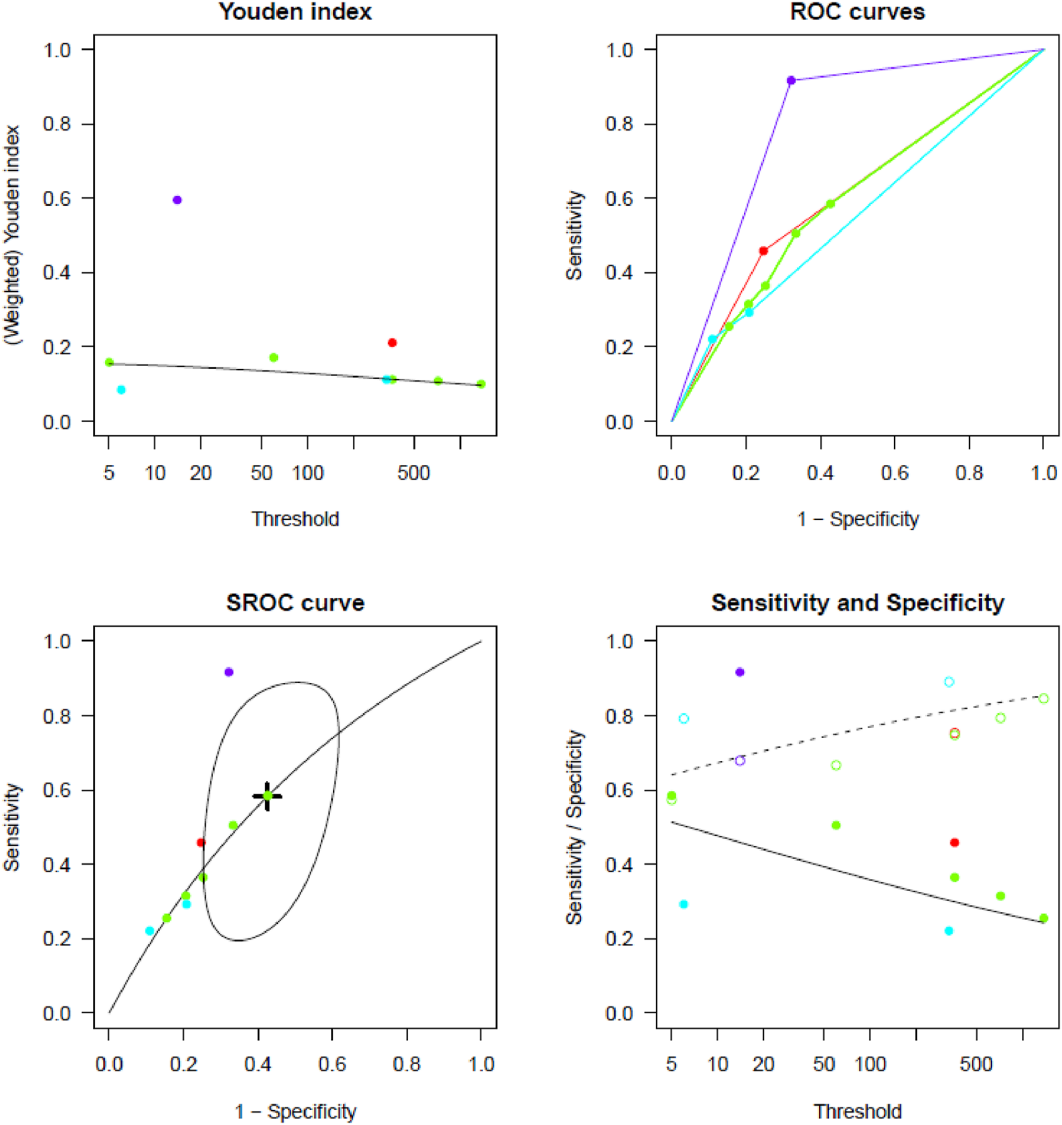
Meta-analysis of diagnostic test accuracy studies with multiple burden cut-offs assessing the differential performances of various cumulative AHRE burden thresholds in predicting thromboembolic events. (**A**) Youden index as a function of test thresholds; (**B**) study-specific ROC curves; (**C**) meta-analytic SROC curve; (**D**) meta-analytic sensitivity/specificity as a function of different test thresholds.

**Figure 4:**
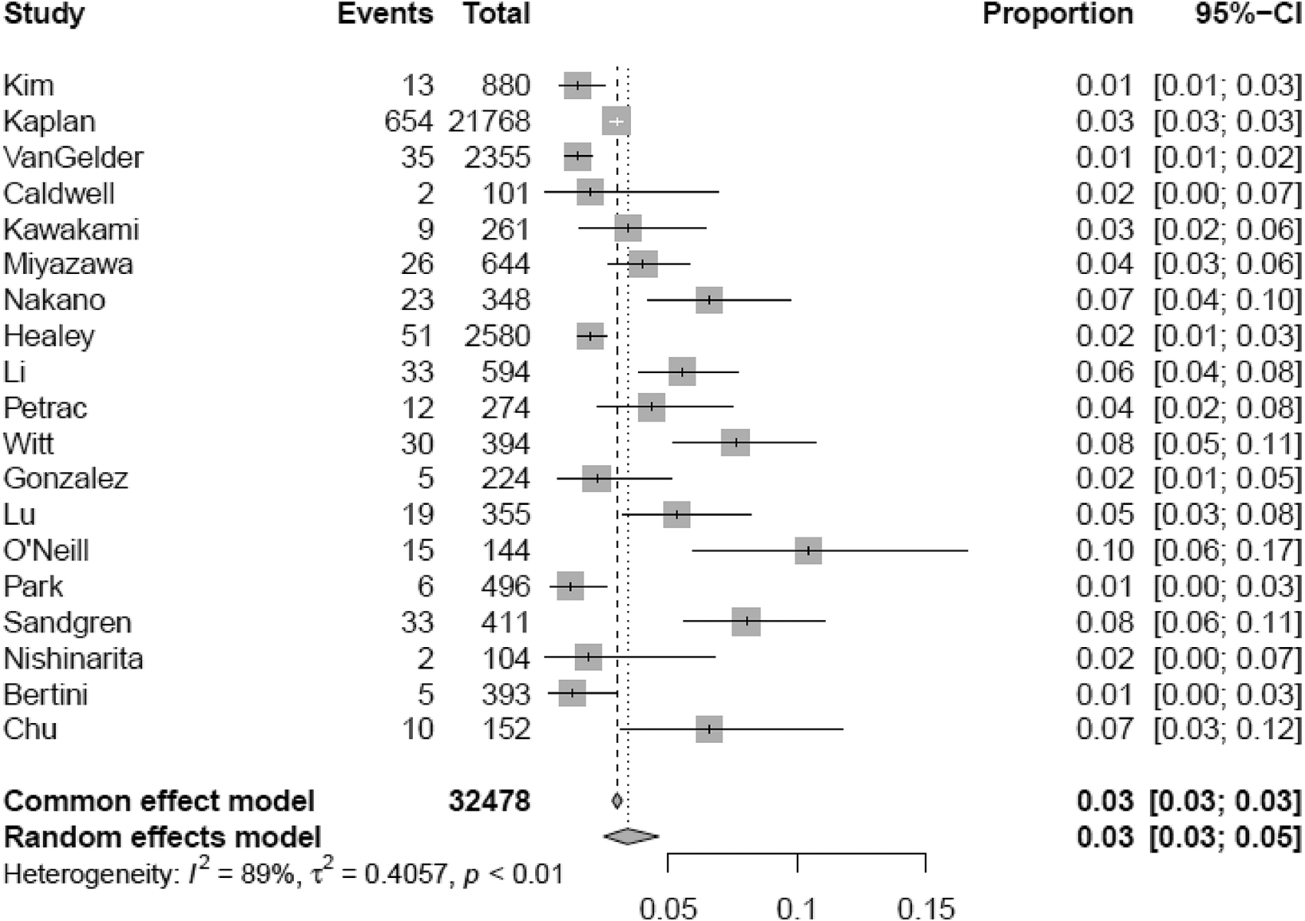
Meta-analytic forest plot (random effects model) reporting the prevalence of thromboembolic events in patients with CIED based on the single longest AHRE duration.

**Figure 5:**
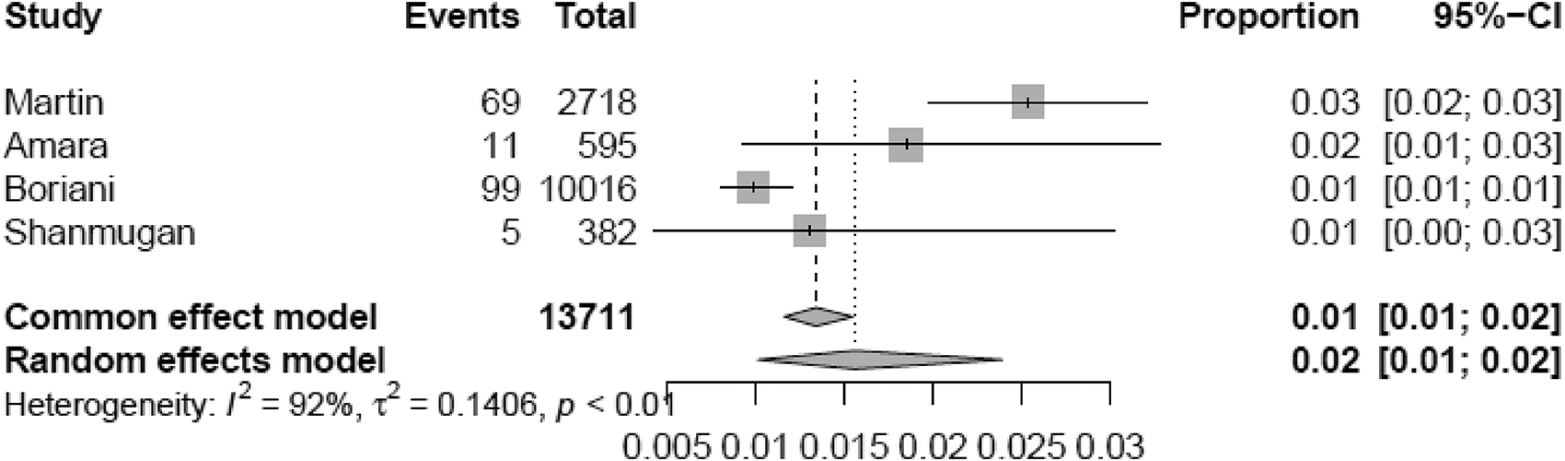
Meta-analytic forest plot (random effects model) reporting the prevalence of thromboembolic events in patients with CIED based on cumulative AHRE burden.

Figures 2, 3 report the Youden index as a function of test thresholds (panel a), study-specific ROC curves (panel b), the resulting meta-analytic SROC curve (panel c), and the meta-analytic sensitivity/specificity as a function of different test thresholds (panel d) for the analyses on single longest and cumulative AHRE burden, respectively. Figures 4, 5 report the meta-analytic forest plots assessing the prevalence of thromboembolic events in patients with single longest and cumulative AHRE burden, respectively.

For the single longest AHRE episode above the optimal meta-analytic threshold (0.07 min), PPV and NPV were 4.7% and 97.7%, respectively. For a cumulative AHRE burden above the optimal meta-analytic threshold (1.4 min), PPV and NPV were 2.1% and 98.9%, respectively.

During the sensitivity analysis considering studies reporting data on patients without previous AF (16 studies all analyzing single longest AHRE duration: total number of cut-offs 29, number of different cut-offs 10), 0.3 min emerged as the optimal AHRE duration. In this subgroup, the sensitivity and specificity at optimal cut-offs resulted in 67.8% (95% CI 46.2%–83.9%) and 62.3% (95% CI 53.2%–70.6%), respectively, with an AUC-SROC of 0.70 (Supplementary Figure S1). PPV and NPV (Supplementary Figure S2) were 6.2% and 98.2%, respectively. After including high-quality studies only (15 studies, all analyzing single longest AHRE), the results were similar to those of the primary analysis (optimal AHRE duration of 0.07 min; sensitivity and specificity of 64.9% and 52.9%, respectively; AUC-SROC: 0.62; Supplementary Figure S3). PPV and NPV are 4.7% and 97.7%, respectively (Supplementary Figure S4).

## Discussion

The main findings of the present meta-analysis (Graphical Abstract) are the following:

• Thromboembolic events are not frequent in the CIED population, and the presence of AHRE does not extensively increase this risk (suboptimal PPV).
• In patients with CIED, the thromboembolic risk poorly relates to AHRE duration, both in terms of the longest single episode and cumulative burden (overall modest AUC-SROC).

Although the topic has been extensively discussed for several years, an evidence-based strategy to manage AHRE in CIED patients is lacking. Even terminologies have been inconsistent: the 2016 ESC guidelines on AF defined AHRE as atrial episodes at a rate >180 bpm lasting more than 5–6 min (5); the 2017 HRS/EHRA/ECAS/APHRS/SOLAECE Consensus included any event, independent of duration, faster than 190 bpm (6); and eventually, the 2020 ESC AF guidelines update stated that the AHRE definition is “usually set” at atrial rate episodes ≥175 bpm lasting more than 5 min (7).

The thromboembolic risk of CIED patients with AHRE differs from that of subjects presenting clinical AF (37). Therefore, in this setting, the need for anticoagulation is poorly defined and must be carefully balanced against the hemorrhagic risk (38). Current guidelines recommend against the routine use of oral anticoagulants in patients with AHRE, suggesting their use only in selected patients with long episodes (>24 h) and high clinical thromboembolic risk (7, 20, 22). Nevertheless, the 24 h cut-off is based mainly on experts’ consensus, not high-grade evidence. A recent meta-analysis by Sagris et al. (12) confirmed previous findings, showing that an AHRE longer than 30 s and a cumulative burden of more than 24 h related to an increased risk. However, a linear relationship between AHRE duration and thromboembolic events was not observed. A new cut-off time for AHRE duration was not proposed nor investigated. A previous report from the RATE registry (39) failed to find a significant association between very short atrial tachycardia episodes detected with CIED interrogation and clinical events or progression to clinical arrhythmia. However, these findings were limited by the fact that short episodes were defined as episodes limited to a single electrogram recording without a clear temporal definition, including any atrial tachycardia of more than three consecutive premature atrial complexes.

Since AHREs are a dynamic entity, which might progress to longer duration and higher burden (40), a stricter follow-up is surely advocated to identify clinical or symptomatic AF. Interestingly, a sub-analysis of the IMPACT trial (26) showed that the duration of AHRE correlates with comorbidities, concurrent risk factors, and CHA_2_DS_2_-VASc score, with a higher percentage of patients with clinical AF or atrial flutter in those with AHRE longer than 24 h. Conversely, previous studies (41, 42) did not demonstrate a clear temporal association between AHRE and stroke, suggesting that AHRE likely represents a marker of increased stroke risk rather than a direct cause (9, 43). Therefore, managing AHRE detected at routine device control or outpatient visits remains a frequently incurred clinical challenge for cardiologists.

Arising from the aforementioned contradictory background, this meta-analysis aims to identify an ideal temporal cut-off to define clinically significant AHRE. To the best of our knowledge, this is the first analysis specifically focused on establishing a clear temporal definition of significant AHRE based on all available evidence. The single longest episode analysis advocates that the optimal threshold tends toward very short episodes (0.07 min), suggesting that the presence determines an increase in the thromboembolic risk more than the duration of the specific AHRE. Similarly, the optimal cut-off identified by the model ran on cumulative AHRE burden also identified a short time frame (1.4 min). Since these limits were statistically derived from the distribution of duration values in the model, they should not be considered new cut-offs for implementation in clinical practice. Instead, the key point is that cut-off points are brief, suggesting that the duration of the atrial episodes poorly relates to the thromboembolic risk. Interestingly, when examining PPV and NPV at different AHRE cut-offs (Table 2 and Graphical Abstract), no substantial difference in thromboembolic risk can be appreciated between longer and shorter episodes. Moreover, the consequent ischemic risk was not extensively higher than that at baseline.

**Table 2:** Sensitivity, specificity, PPV, and NPV at different cut-offs.

Cut-off	Prevalence	Sensitivity (95% CI)	Specificity (95% CI)	PPV	NPV
Longest AHRE					
6 min	3.4%	36.1% (23.2%–51.3%)	77.0% (69.9%–82.8%)	5.3%	97.1%
24 h	3.4%	11.4% (5.7%–21.5%)	92.8% (88.6%–95.5%)	5.3%	96.7%
Cumulative AHRE burden					
6 min	1.6%	50.4% (25.1%–75.5%)	64.9% (50.8%–76.8%)	2.2%	98.8%
24 h	1.6%	24.1% (10.6%–46.1%)	85.5% (79.0%–90.2%)	2.6%	98.6%
Patients without prior AF (longest AHRE)					
6 min	3.5%	47.7% (27.7%–68.6%)	78.2% (69.6%–84.9%)	7.4%	97.6%
24 h	3.5%	15.8% (6.0%–35.4%)	94.0% (89.2%–96.7%)	8.7%	96.8%
High-quality studies only (longest AHRE)					
6 min	3.4%	35.8% (22.6%–51.6%)	77.0% (68.3%–84.0%)	5.3%	97.1%
24 h	3.4%	11.4% (5.4%–22.4%)	92.8% (87.5%–95.9%)	5.3%	96.7%

Therefore, these findings suggest that AHRE may be considered a marker of atrial cardiomyopathy (44, 45), a complex derangement of the anatomic, electrical, and physiological features of the atrial chamber. Atrial cardiomyopathy correlates, at least partially, with an increased risk of stroke, independent of the underlying heart rhythm. The need for OAC in this setting must be confirmed by a prospective and randomized study. Most likely, other elements (e.g., left atrial size, NT-proBNP levels, and CHA_2_DS_2_-VASc score) must be considered to guide the initiation of anticoagulation in patients with AHRE. From this perspective, the present analysis supports this theory and should be regarded as hypothesis-generating for future investigations, aiming to identify a common AHRE temporal cut-off to be used in the design of prospective studies.

Altogether, the present analysis documents that neither the longest single AHRE duration nor the cumulative burden is linearly associated with the thromboembolic risk, supporting the hypothesis that any documented AHRE constitutes an additional element in assessing patient-specific thromboembolic risks. These results suggest that short AHRE episodes might not indicate *per se* the need for anticoagulation but should be considered in evaluating the clinical risk of a patient. In fact, the recently published NOAH-AFNET trial supports these results by indicating that a careful assessment of the global risk is necessary, as the bleeding risk of the anticoagulation therapy may offset the beneficial protection from ischemic events (46). Nevertheless, further studies, including the termination of the ARTESiA (47) trial, will shed further light on the topic.

### Limitations

The following limitations need to be addressed. First, the between-study variations in AHRE definitions as well as end-point identifications might have partially affected the relationship between AHRE and thromboembolic events. Second, sensitivity analyses on patients without previous history of AF or including high-quality studies only could not be performed on cumulative AHRE burden analysis due to the limited number of studies reporting multiple cut-offs. Although AHRE refers to asymptomatic patients without prior AF, patients with AF history were not censored in the primary analysis since this small proportion of patients was considered in most of the included studies. In addition, a small percentage of patients were on OAC. Given that this may affect the incidence of thromboembolic events, as emerged in the sensitivity analysis on patients without prior AF, OACs were also prescribed for conditions other than AF, representing routine clinical practice well. Finally, the present meta-analysis was not based on individual patient data, and the included studies exhibit heterogeneity in methods, populations, and assessments of outcomes.

## Conclusions

The detection of AHRE in CIED patients is a frequent clinical challenge. This study aims to evaluate the thromboembolic risk of patients with CIED and AHRE, without implying a recommendation for using OACs in this patient cohort. Based on the present diagnostic meta-analysis with multiple cut-offs, it emerges that presence, rather than AHRE duration, confers an increased, albeit low, thromboembolic risk. In the absence of a linear relationship between AHRE duration (both in terms of single longest episode than cumulative burden) and thromboembolic events, any AHRE reported in CIED patients should constitute an additional element in assessing patient-specific thromboembolic risks.

## Data Availability

The original contributions presented in the study are included in the article/**Supplementary Material**, further inquiries can be directed to the corresponding author.

## References

[1] Lamas GA Lee KL Sweeney MO Silverman R Leon A Yee R Ventricular pacing or dual-chamber pacing for sinus-node dysfunction. N Engl J Med. (2002) 346:1854–62. 10.1056/NEJMoa013040 12063369

[2] Healey JS Connolly SJ Gold MR Israel CW Van Gelder IC Capucci A Subclinical atrial fibrillation and the risk of stroke. N Engl J Med. (2012) 366:120–9. 10.1056/nejmoa1105575 22236222

[3] Ziegler PD Glotzer T V Daoud EG Singer DE Ezekowitz MD Hoyt RH Detection of previously undiagnosed atrial fibrillation in patients with stroke risk factors and usefulness of continuous monitoring in primary stroke prevention. Am J Cardiol. (2012) 110:1309–14. 10.1016/j.amjcard.2012.06.034 22819433

[4] Boriani G Vitolo M Imberti JF Potpara TS Lip GYH. What do we do about atrial high rate episodes? Eur Heart J Suppl. (2020) 22:O42–52. 10.1093/EURHEARTJ/SUAA179 33380943 PMC7753882

[5] Kirchhof P Benussi S Kotecha D Ahlsson A Atar D Casadei B 2016 ESC guidelines for the management of atrial fibrillation developed in collaboration with EACTS. Eur J Cardiothorac Surg. (2016) 5:e81–8. 10.1093/ejcts/ezw313 27663299

[6] Calkins H Hindricks G Cappato R Kim Y-H Saad EB Aguinaga L 2017 HRS/EHRA/ECAS/APHRS/SOLAECE expert consensus statement on catheter and surgical ablation of atrial fibrillation. Europace. (2018) 20:e1–160. 10.1093/europace/eux274 29016840 PMC5834122

[7] Hindricks G Potpara T Dagres N Arbelo E Bax JJ Blomström-Lundqvist C 2020 ESC guidelines for the diagnosis and management of atrial fibrillation developed in collaboration with the European Association for Cardio-Thoracic Surgery (EACTS). Eur Heart J. (2021) 42:373–498. 10.1093/eurheartj/ehaa612 32860505

[8] Gorenek B Bax J Boriani G Chen SA Dagres N Glotzer T V Device-detected subclinical atrial tachyarrhythmias: definition, implications and management—an European Heart Rhythm Association (EHRA) consensus document, endorsed by Heart Rhythm Society (HRS), Asia Pacific Heart Rhythm Society (APHRS) and Sociedad L. Europace. (2017) 19:1556–78. 10.1093/europace/eux163 28934408

[9] Freedman B Boriani G Glotzer TV Healey JS Kirchhof P Potpara TS. Management of atrial high-rate episodes detected by cardiac implanted electronic devices. Nat Rev Cardiol. (2017) 14:701–14. 10.1038/nrcardio.2017.94 28682320

[10] Saglietto A Ballatore A Xhakupi H Rubat Baleuri F Magnano M Gaita F Evidence-based insights on ideal blanking period duration following atrial fibrillation catheter ablation. EP Eur. (2022) 24:1899–908. 10.1093/EUROPACE/EUAC098 35917218

[11] Steinhauser S Schumacher M Rücker G. Modelling multiple thresholds in meta-analysis of diagnostic test accuracy studies. BMC Med Res Methodol. (2016) 16:97. 10.1186/s12874-016-0196-1 27520527 PMC4983029

[12] Sagris D Georgiopoulos G Pateras K Perlepe K Korompoki E Milionis H Atrial high-rate episode duration thresholds and thromboembolic risk: a systematic review and meta-analysis. J Am Heart Assoc. (2021) 10:e022487. 10.1161/JAHA.121.022487 34755543 PMC8751956

[13] Ouzzani M Hammady H Fedorowicz Z Elmagarmid A. Rayyan—a web and mobile app for systematic reviews. Syst Rev. (2016) 5(1):210. 10.1186/S13643-016-0384-4 27919275 PMC5139140

[14] Rücker G Steinhauser S Kolampally S Schwarzer G. Diagmeta: meta-analysis of diagnostic accuracy studies with several cutpoints. R package version 0.5-1 (2022). Available at: https://CRAN.R-project.org/package=diagmeta

[15] Bertini M Borleffs CJW Delgado V Ng ACT Piers SRD Shanks M Prediction of atrial fibrillation in patients with an implantable cardioverter–defibrillator and heart failure. Eur J Heart Fail. (2010) 12:1101–10. 10.1093/eurjhf/hfq126 20861134

[16] Nishinarita R Niwano S Fukaya H Oikawa J Nabeta T Matsuura G Burden of implanted-device-detected atrial high-rate episode is associated with future heart failure events—clinical significance of asymptomatic atrial fibrillation in patients with implantable cardiac electronic devices. Circ J. (2019) 83:736–42. 10.1253/circj.CJ-18-1130 30814400

[17] Petrač D Radeljić V Delić-Brkljačić D Manola Š Cindrić-Bogdan G Pavlović N. Persistent atrial fibrillation is associated with a poor prognosis in patients with atrioventricular block and dual-chamber pacemaker. Pacing Clin Electrophysiol. (2012) 35:695–702. 10.1111/j.1540-8159.2012.03376.x 22452373

[18] Kim BS Chun KJ Hwang JK Park SJ Park KM Kim JS Predictors and long-term clinical outcomes of newly developed atrial fibrillation in patients with cardiac implantable electronic devices. Medicine (Baltimore) (2016) 95:e4181. 10.1097/MD.0000000000004181 27428213 PMC4956807

[19] Kaplan RM Koehler J Ziegler PD Sarkar S Zweibel S Passman RS. Stroke risk as a function of atrial fibrillation duration and CHA_2_DS_2_-VASc score. Circulation. (2019) 140:1639–46. 10.1161/CIRCULATIONAHA.119.041303 31564126

[20] Van Gelder IC Healey JS Crijns HJGM Wang J Hohnloser SH Gold MR Duration of device-detected subclinical atrial fibrillation and occurrence of stroke in ASSERT. Eur Heart J. (2017) 38:1339–44. 10.1093/eurheartj/ehx042 28329139

[21] Amara W Montagnier C Cheggour S Boursier M Gully C Barnay C Early detection and treatment of atrial arrhythmias alleviates the arrhythmic burden in paced patients: the SETAM study. Pacing Clin Electrophysiol. (2017) 40:527–36. 10.1111/pace.13062 28244117

[22] Boriani G Glotzer TV Santini M West TM De Melis M Sepsi M Device-detected atrial fibrillation and risk for stroke: an analysis of >10 000 patients from the SOS AF project (Stroke preventiOn Strategies based on Atrial Fibrillation information from implanted devices). Eur Heart J. (2014) 35:508–16. 10.1093/eurheartj/eht491 24334432 PMC3930873

[23] Caldwell JC Contractor H Petkar S Ali R Clarke B Garratt CJ Atrial fibrillation is under-recognized in chronic heart failure: insights from a heart failure cohort treated with cardiac resynchronization therapy. Europace. (2009) 11:1295–300. 10.1093/europace/eup201 19648586

[24] Shanmugam N Boerdlein A Proff J Ong P Valencia O Maier SKG Detection of atrial high-rate events by continuous home monitoring: clinical significance in the heart failure—cardiac resynchronization therapy population. Europace. (2012) 14:230–7. 10.1093/europace/eur293 21933802 PMC3262405

[25] Kawakami H Nagai T Saito M Inaba S Seike F Nishimura K Clinical significance of atrial high-rate episodes for thromboembolic events in Japanese population. Heart Asia. (2017) 9:e010954. 10.1136/heartasia-2017-010954 29177015 PMC5692098

[26] Miyazawa K Pastori D Martin DT Choucair WK Halperin JL Lip GYH. Characteristics of patients with atrial high rate episodes detected by implanted defibrillator and resynchronization devices. Europace. (2022) 24:375–83. 10.1093/europace/euab186 34426836 PMC8892042

[27] Nakano M Kondo Y Nakano M Kajiyama T Hayashi T Ito R Impact of atrial high-rate episodes on the risk of future stroke. J Cardiol. (2019) 74:144–9. 10.1016/j.jjcc.2019.01.006 30728105

[28] Li YG Miyazawa K Pastori D Szekely O Shahid F Lip GYH. Atrial high-rate episodes and thromboembolism in patients without atrial fibrillation: the West Birmingham Atrial Fibrillation Project. Int J Cardiol. (2019) 292:126–30. 10.1016/j.ijcard.2019.04.055 31031080

[29] Witt CT Kronborg MB Nohr EA Mortensen PT Gerdes C Nielsen JC. Early detection of atrial high rate episodes predicts atrial fibrillation and thromboembolic events in patients with cardiac resynchronization therapy. Heart Rhythm. (2015) 12:2368–75. 10.1016/j.hrthm.2015.07.007 26164377

[30] Gonzalez M Keating RJ Markowitz SM Liu CF Thomas G Ip JE Newly detected atrial high rate episodes predict long-term mortality outcomes in patients with permanent pacemakers. Heart Rhythm. (2014) 11:2214–21. 10.1016/j.hrthm.2014.08.019 25131667

[31] Da LW Chen JY. The optimal cutoff of atrial high-rate episodes for neurological events in patients with dual chamber permanent pacemakers. Clin Cardiol. (2021) 44:871–9. 10.1002/clc.23626 34002855 PMC8207987

[32] O’Neill J Jegodzinski L Tayebjee MH. Incidence of subclinical atrial fibrillation in a South Asian population. Pacing Clin Electrophysiol. (2018) 41:1600–5. 10.1111/pace.13516 30267542

[33] Park YJ Kim JS Park KM On YK Park SJ. Subclinical atrial fibrillation burden and adverse clinical outcomes in patients with permanent pacemakers. Stroke. (2021) 52:1299–308. 10.1161/STROKEAHA.120.031822 33588601

[34] Sandgren E Rorsman C Edvardsson N Engdahl J. Stroke incidence and anticoagulation treatment in patients with pacemaker-detected silent atrial fibrillation. PLoS One. (2018) 13:1–12. 10.1371/journal.pone.0203661 PMC6136732 30212562

[35] Chu SY Jiang J Wang YL Sheng QH Zhou J Ding YS. Pacemaker-detected atrial fibrillation burden and risk of ischemic stroke or thromboembolic events—a cohort study. Heart Lung. (2020) 49:66–72. 10.1016/j.hrtlng.2019.07.007 31376922

[36] Martin DT Bersohn MM Waldo AL Wathen MS Choucair WK Lip GYH Randomized trial of atrial arrhythmia monitoring to guide anticoagulation in patients with implanted defibrillator and cardiac resynchronization devices. Eur Heart J. (2015) 36:1660–8. 10.1093/eurheartj/ehv115 25908774

[37] Mahajan R Perera T Elliott AD Twomey DJ Kumar S Munwar DA Subclinical device-detected atrial fibrillation and stroke risk: a systematic review and meta-analysis. Eur Heart J. (2018) 39:1407–15. 10.1093/eurheartj/ehx731 29340587

[38] Bertaglia E Blank B Blomström-Lundqvist C Brandes A Cabanelas N Dan GA Atrial high-rate episodes: prevalence, stroke risk, implications for management, and clinical gaps in evidence. Europace. (2019) 21:1459–67. 10.1093/EUROPACE/EUZ172 31377792 PMC6788209

[39] Swiryn S Orlov MV Benditt DG Dimarco JP Lloyd-Jones DM Karst E Clinical implications of brief device-detected atrial tachyarrhythmias in a cardiac rhythm management device population: results from the Registry of Atrial Tachycardia and Atrial Fibrillation Episodes. Circulation. (2016) 134:1130–40. 10.1161/CIRCULATIONAHA.115.020252 27754946

[40] Boriani G Glotzer T V Ziegler PD De Melis M Mangoni di S. Stefano L Sepsi M Detection of new atrial fibrillation in patients with cardiac implanted electronic devices and factors associated with transition to higher device-detected atrial fibrillation burden. Heart Rhythm. (2018) 15:376–83. 10.1016/j.hrthm.2017.11.007 29122724

[41] Daoud EG Glotzer T V Wyse DG Ezekowitz MD Hilker C Koehler J Temporal relationship of atrial tachyarrhythmias, cerebrovascular events, and systemic emboli based on stored device data: a subgroup analysis of TRENDS. Heart Rhythm. (2011) 8:1416–23. 10.1016/j.hrthm.2011.04.022 21699833

[42] Brambatti M Connolly SJ Gold MR Morillo CA Capucci A Muto C Temporal relationship between subclinical atrial fibrillation and embolic events. Circulation. (2014) 129:2094–9. 10.1161/CIRCULATIONAHA.113.007825 24633881

[43] Camm AJ Simantirakis E Goette A Lip GYH Vardas P Calvert M Atrial high-rate episodes and stroke prevention. Europace. (2017) 19:169–79. 10.1093/europace/euw279 28172715 PMC5400077

[44] Kamel H Okin PM Longstreth WT Elkind MSV Soliman EZ. Atrial cardiopathy: a broadened concept of left atrial thromboembolism beyond atrial fibrillation. Future Cardiology. (2015) 11:323–31. 10.2217/FCA.15.22 26021638 PMC4868349

[45] Kamel H Okin PM Elkind MSV Iadecola C. Atrial fibrillation and mechanisms of stroke. Stroke. (2016) 47:895–900. 10.1161/STROKEAHA.115.012004 26786114 PMC4766055

[46] Kirchhof P Toennis T Goette A Camm AJ Diener HC Becher N Anticoagulation with edoxaban in patients with atrial high-rate episodes. N Engl J Med. (2023) 389:1167–79. 10.1056/NEJMOA2303062/SUPPL_FILE/NEJMOA2303062_DATA-SHARING.PDF 37622677

[47] Lopes RD Alings M Connolly SJ Beresh H Granger CB Mazuecos JB Rationale and design of the apixaban for the reduction of thrombo-embolism in patients with device-detected sub-clinical atrial fibrillation (ARTESiA) trial. Am Heart J. (2017) 189:137–45. 10.1016/j.ahj.2017.04.008 28625370

