# Supplementary material for: Role of atrial high-rate episodes in stratifying thromboembolic risk: a multiple cut-off diagnostic meta-analysis
Source: Front Cardiovasc Med. 2023 Nov 7;10:1289372. doi: 10.3389/fcvm.2023.1289372 (PMC10662047; doi:10.3389/fcvm.2023.1289372)
Supplement: Supplementary file 1 [file Table1.docx]

**Role of atrial high-rate episodes in stratifying thromboembolic risk: a multiple cut-off diagnostic meta-analysis**

*Supplementary Material*

**Study selection**

We here provide a detailed description of the study selection process.

33 studies were identified from examination of relevant literature on the topic. Of these, 28 were not retrieved because duplicates, already present in the results of database search or not relevant from title and abstract screening. 5 studies were assessed for eligibility, of which 2 studies^1,2^ were excluded and 3^3–5^ were finally included in the analysis.

10’188 records were identified from database search. Rayyan.ai semi-automatic tool was used for the first screening phase. 1’024 studies were manually analyzed to train the algorithm, subsequently all records with the lowest rating for inclusion (half a star out of five) were automatically excluded. 10’058 were excluded through screening of title and abstract, of which 2’932 were excluded by use of semi-automatic tools, and 130 were finally assessed for eligibility. Of these, 110 were excluded because they were duplicates, they did not comply with the inclusion criteria or they lacked count data on thromboembolic events associated with AHRE.

**Supplementary Tables**

**Table S1.** Quality assessment of the included studies (Newcastle-Ottawa scale)

| **Study, year** | **Selection** | **Comparability** | **Outcome** | **Total** |
| --- | --- | --- | --- | --- |
| Kim, 2016^6^ | **** | ** | ** | 8/9 |
| Kaplan, 2019^7^ | *** | ** | ** | 7/9 |
| VanGelder, 2017^8^ | *** | ** | *** | 8/9 |
| Amara, 2017^9^ | **** |  | *** | 7/9 |
| Boriani, 2014^10^ | *** | ** | ** | 7/9 |
| Caldwell, 2009^11^ | *** |  | ** | 5/9 |
| Shanmugan, 2012^12^ | *** | ** | *** | 8/9 |
| Kawakami, 2017^13^ | *** | ** | *** | 8/9 |
| Miyazawa, 2019^14^ | **** | ** | ** | 8/9 |
| Nakano, 2019^15^ | **** | ** | ** | 8/9 |
| Healey, 2012^16^ | *** | ** | *** | 8/9 |
| Li, 2019^17^ | *** | ** | ** | 7/9 |
| Petrac, 2012^5^ | *** |  | *** | 6/9 |
| Witt, 2015^18^ | *** | ** | *** | 8/9 |
| Gonzalez, 2014^19^ | **** | ** | ** | 8/9 |
| Lu, 2021^20^ | *** | ** | *** | 8/9 |
| O’Neill, 2018^21^ | *** | ** | ** | 7/9 |
| Park, 2021^22^ | *** | ** | *** | 8/9 |
| Sandgren, 2018^23^ | *** |  | *** | 6/9 |
| Nishinarita, 2019^4^ | *** | ** | *** | 8/9 |
| Chu, 2020^24^ | ** | ** | ** | 7/9 |
| Martin, 2015^25^ | ** | * | *** | 6/9 |
| Bertini, 2010^3^ | ** | ** | ** | 6/9 |

**Table S2.** Characteristics of the included studies.

| Study Name | **Kim 2016** | **Kaplan 2019** | **Van Gelder 2017 (ASSERT)** | **Amara 2017 (SETAM)** | **Caldwell 2009** | **O’Neill 2018** |
| --- | --- | --- | --- | --- | --- | --- |
| General characteristics | Retrospective, single centre | Retrospective, multicentre | Prospective, multicentre | Prospective, randomized, multicentre | Retrospective, single centre | Retrospective, single centre |
| Patient population | Patients with CIED and no history of AF | Patients with CIED and no anticoagulation therapy | Patients with CIED, > 65 years, with hypertension and no history of AF | Patients with CIED and no history of AT | Patients with CHF treated with CRT | Patients with CIED and no history of AT |
| Number of patients | 880 | 21,768 | 2455 | 595 | 162 (101 no history of AF) | 144 |
| Number of patients with AHRE/subclinical AF | 122 | 4953 (AF > 6 min)  1572 (AF > 23.5 hours) | 893 (AF > 6 min)  431 (AF > 6 hours)  262 (AF > 24 hours) | 149 | 27 | 18 |
| Year of publication | 2016 | 2019 | 2017 | 2017 | 2009 | 2018 |
| ICM device | PM, ICD and CRT | Dual chamber PM, ICD and CRT | Dual chamber PM, ICD | PM | CRT | Dual chamber PM, CRT |
| OAC use at baseline | 4.5% | 0% | 0% | 0% | 13.5% | 2.8% |
| Follow-up monitoring strategy | Follow-up visits at 6-month (PM) and 3-month (ICD/CRT) intervals with device interrogation by 2 electrophysiologists blinded to the patients’ clinical events. | Clinical data derived from electronic health record. CIED data on AF burden from Medtronic CareLink database. | First follow-up visit at 3 month and subsequently at 6-months interval. | Follow-up visits at 1-3 months and at 12-months.  Daily monitoring in the group of patients with remote monitoring activated, with re-scheduling of the next follow-up visit if necessary. | Routine follow-up for HF with device interrogation | Routine follow-up visits with device interrogation. |
| AHRE/subclinical AF definition | Any AT with an atrial rate ≥ 180 beats/min lasting for at least 5 minutes (in dual chamber CIED). Device-based diagnostic for single chamber CIED. | AT/AF lasting for at least 6 minutes | Any AT with an atrial rate ≥ 190/min lasting for at least 6 minutes (required EGM confirmation) | Any AT with an atrial rate ≥ 200 beats/min lasting for at least 6 hours (required EGM or surface ECG confirmation). | Any mode-switch occurrence on the device with an atrial rate ≥ 200 bpm lasting for at least 30 seconds. | Any AT with an atrial rate > 190 beats/min, lasting for at least 6 minutes. |
| Major events definition | Stroke readmissions: readmissions with a primary diagnosis of cerebral infarction with rapid-onset focal neurologic symptoms lasting at least 24 hours. | Ischemic stroke defined by ICD (9th and 10th revisions) codes and additional clinical criteria for treatment (e.g. mechanical thrombectomy or systemic thrombolysis)  Systemic embolism defined by ICD (9th and 10th revisions) codes. | Ischemic stroke confirmed by imaging  Systemic embolism documented with the use of imaging, surgery or autopsy. | Ischemic stroke | Thromboembolic complications (stroke, TIA, peripheral thromboembolism) | Cerebrovascular events defined as a neurology clinic or hospital discharge diagnosis of stroke or TIA. |
| Average FU | 55.2 months | NA | 30 months | 12.8 months | 14.1 months | 39.4 months |
| Major inclusion criteria | - Age ≥ 18 - At least 1 follow-up visit and device interrogation after implantation. | - Optum EHR and Medtronic CareLink data available, with a cardiovascular diagnosis code in medical records. - Cardiovascular-related procedure performed during the data-collection period. | - Age ≥ 65. - History of hypertension requiring medical therapy. | - CHA2DS2VASC score ≥ 2. - In sinus rhythm when enrolled. - Written informed consent | - NYHA class III or IV chronic heart failure, treated with CRT. - Regular follow-up. | - De novo dual-chamber PM or CRT-P implantations |
| Major exclusion criteria | - Reimplantation of device. - History of documented AF/AFL | - Absence of at least 12 months of electronic health record data before the index date. | - History of AF. - Treatment with VKA | - Treatment with a class I or III antiarrythmic drug, DAPT or long-term anticoagulation, or contraindications to these treatments - Previous history of documented atrial arrythmias. | NA | - History of atrial arrhythmia. - CIED follow-up performed at a different center. - No atrial lead. - Congenital heart disease. |
| Primary endpoint | Composite of mortality and unplanned cardiovascular readmission. | Composite of ischemic stroke and systemic embolism. | Composite of ischemic stroke and systemic embolism. | Effects of remote monitoring on detection of AT. | Mortality, hospitalization, thromboembolic complications. | Incidence of subclinical AF. |
| Secondary endpoints | Mortality, stroke readmissions, HF readmissions, other cardiovascular readmissions, unplanned cardiovascular readmission | NA | NA | AT burden and incidence.  AT-related adverse clinical events. | NA | Incidence of clinical AF, cerebrovascular events, and all-cause mortality. |
| Sponsor | NA | American Heart Association | St Jude Medical | Biotronik SE | NIHR Biomedical Research Centre  Medtronic Inc | NA |

| Study Name | **Boriani 2014 (TRENDS, PANORAMA, Italian ClinicalService)** | **Shanmugam 2011**  **(HomeCARE and everesT trials)** | **Kawakami 2017** | **Healey 2012**  **(ASSERT)** | **Nishinarita 2019** | **Petrac 2012** |
| --- | --- | --- | --- | --- | --- | --- |
| General characteristics | Prospective, multicentre. (pooled analysis) | Prospective, multicentre. | Retrospective, multicentre. | Prospective, multicentre. | Retrospective, single centre. | Retrospective, single centre |
| Patient population | Patients with CIED and no history of permanent AF | HF patients with CRT and active home monitoring | Patients with CIED and no history of PerAF or permanent AF | Patients with CIED > 65 years with hypertension and no history of AF | Patients with CIED and no history of AF | Patients with dual chamber PM for II- or III-degree AV block and no history of AF. |
| Number of patients | 10,016 | 560 (382 no history of previous FA) | 343 (261 no previous PAF) | 2,580 | 104 | 308 |
| Number of patients with AHRE | 4287 (AF > 5 min)  3355 (AF > 1 h)  2541 (AF > 6 h)  2076 (AF > 12 h)  1556 (AF > 23 h) | 126 | 165 (96 no previous PAF) | 261 | 34 | 76 |
| Year of publication | 2014 | 2011 | 2017 | 2012 | 2019 | 2012 |
| ICM device | PM, ICD, CRT | CRT | Dual chamber PM | Dual chamber PM, ICD | Dual chamber PM | Dual chamber PM |
| OAC use at baseline | 18% | 8.1% | 15% | 0% | 10.2% | 1% |
| Follow-up monitoring strategy | TRENDS: device interrogation every 3-months; clinical visit every 6-months.  PANORAMA and the Italian ClinicalService Project: follow-up visits with clinical check and device interrogation according to the routine practice of the participating centres. | Home monitoring was activated in all devices. | Follow-up visits at 3/6-month interval with clinical check and device interrogation. | First follow-up visit at 3 month and subsequently at 6-months interval. | Initial device interrogation 1 week after implantation. Clinical visit and device interrogation every 6-months | Follow up visits at 6-month interval for a minimum of two years. |
| AHRE/subclinical AF definition | Maximum daily burden detected by the device (at least 5 minutes). | Total daily duration of mode-switch (at least 14 min) | Any AT with an atrial rate > 175 bpm lasting for at least 6 minutes. | Any AT with an atrial rate > 190 bpm lasting for more than 6 minutes (required EGM confirmation). | Any AT with an atrial rate > 170 bpm lasting for more than 5 minutes (required EGM confirmation). | Any AT with an atrial rate > 220 bpm lasting for at least 5 minutes. |
| Major events definition | Ischemic stroke and TIA defined by the judgement of neurologist | Ischemic stroke, TIA, peripheral thromboembolism | Ischemic stroke confirmed by imaging and systemic thromboembolism | Ischemic stroke confirmed by imaging  Systemic embolism documented with the use of imaging, surgery or autopsy. | Stroke | Stroke (nonfatal) |
| Average FU | 24 months | 12 months | 52 months | 30 months | 65 months | 36 months |
| Major inclusion criteria | - Implantation of a device capable of measuring atrial tachyarrhythmias. - At least 3 months of follow-up. - Device diagnostic data available. | - HF patient with a CRT device capable of continuous heart rhythm monitoring via HM - Patients in SR in more than 70% HM transmissions in the follow-up period - > 3 months of HM follow-up | - Age ≥ 65 - First implantation | - Age ≥ 65 - History of hypertension - Written informed consent | - First implantation of dual chamber PM | - First implantation of dual chamber PM. |
| Major exclusion criteria | - History of permanent AF | - Planned cardiac surgical procedures within 6 months after enrollment - Life expectancy ≤ 6 months - Pregnant and breast-feeding women - Participation in another clinical study - Age <18 yo - Unwillingness or inability to sign written informed consent | - History of perAF or permanent AF | - History of AF/AFL lasting for more than 5 minutes. - Treatment with a VKA | - Patients with ICD/CRT or PM without atrial lead. - Idiopathic cardiomyopathy - end stage renal disease (with hemodialysis). - History of documented AF. | - History of documented AF. - Evidence of SND. - life expectancy <12 months |
| Primary endpoint | NA | Composite of ischemic stroke, TIA and peripheral thromboembolism | Composite of ischemic stroke and systemic embolism. | Composite of ischemic stroke and systemic embolism. | All-cause death and new-onset or worsening HF | Cardiovascular death. |
| Secondary endpoints |  | CV death, hospitalization for AF or worsening of HF. |  |  | Fatal arrhythmia and/or cerebral infarction. | A first occurrence of hospitalization for heart failure, a first nonfatal stroke, and a first occurrence of PAF episode. |
| Sponsor | Medtronic Inc | Biotronik SE | NA | St Jude Medical | None | NA |

| Study Name | **Li 2019**  **(The West Birmingham Atrial Fibrillation Project)** | **Witt 2015** | **Gonzalez 2014** | **Lu 2021** | **Park 2021** | **Sandgren 2018** |
| --- | --- | --- | --- | --- | --- | --- |
| General characteristics | Prospective, single centre. | Retrospective, single centre. | Retrospective, single centre. | Retrospective, single centre. | Retrospective, single centre. | Retrospective, multicentre. |
| Patient population | Patients with CIED with no history of AF. | Patients with CRT and no history of AF or AFL. | Patients with CIED and no history of AF. | Patients with CIED and no history of AF. | Patients with PM and no history of AF. | Patients with CIED. |
| Number of patients | 594 | 394 | 224 | 355 | 496 | 411 |
| Number of patients with AHRE | 175 | 79 | 39 | 162 (AHRE ≥ 30 s) | 344 | 125 |
| Year of publication | 2019 | 2015 | 2014 | 2021 | 2021 | 2018 |
| ICM device | PM, ICD, CRT | CRT | Dual chamber PM | PM | PM | PM, CRT |
| OAC use at baseline | 0% | 14.2% | 3.1% | 9% | 3.8% |  |
| Follow-up monitoring strategy | Clinical visits and device interrogation at 3-9 months interval with | Clinical visits with device interrogation 1 and 6 months after implantation and subsequently every 6-12 months. | Clinical visits and device interrogation at 1-2 weeks, 3-4 months and then annually after implantation. | Clinical visits and device interrogation at 3/6-month interval. | Clinical visits and device interrogation at 3 months and then every 6 months after implantation. | Routine clinical follow-up |
| AHRE/subclinical AF definition | Any AT with an atrial rate ≥ 175 bpm lasting for at least 5 minutes. | Any AT (according to manufacturer-specific nominal settings) lasting for at least than 6 minutes. | Any AT lasting for at least 5 minutes (cutoff programmed at the discretion of the operator, mean atrial rate 178 $\pm$12 bpm) (required ECG confirmation) AHREs occurring within 48 hours of device implantation were excluded. | Any AT with an atrial rate > 175 bpm (MEDTRONIC) or > 200 bpm (BIOTRONIK) and lasting for at least 30 seconds (required EGM confirmation) | Any AT with an atrial rate > 170 bpm lasting for at least 6 minutes. | Any AF lasting for at least 5 minutes recorded by a device with an AF-detection algorithm or confirmed by EGM |
| Major events definition | Ischemic stroke (confirmed by imaging), TIA, systemic thromboembolism (confirmed by imaging, surgery or autopsy) | Ischemic stroke, TIA, or systemic thromboembolism defined by ICD (10th revisions) codes. | Fatal stroke defined by ICD (9^th^ and 10^th^ revisions) codes. | Ischemic stroke, TIA | Ischemic stroke confirmed by imaging | Ischemic stroke |
| Average FU | 50.4 months | 50 months | 79.2 months | 42.1 months | 62.4 months | 38 months |
| Major inclusion criteria | - Implantation of PM, CRT or ICD | - Patients with standard indication for CRT. | - Implantation of dual-chamber PM | - Age > 18. - Implantation of dual chamber PM Written informed consent | - Age ≥18. - De novo PM implantation based on cur- rent guidelines - PM with atrial sensing capability. - Written informed consent. | - Implantation of dual-chamber PPM or CRT-P for SND or AV/BB block |
| Major exclusion criteria | - History of AF - Anticoagulation therapy. | - History of AF or AFL. - No device interrogation beyond 1 month. - Death or system downgrading to PM or ICD within the 6 month follow up visit. - prosthetic valve. | - Guidant PM. - Missing social security numbers - EGM storage not activated. - PM indication for neurocardiogenic syncope. - History of AF. - Less than 6 months of follow-up. | - History of AF. | - History of documented AF or AFL. - Atrial lead fracture or malfunction making the detection of a true atrial signal impossible or unreliable. - Lack of or insufficient data on SCAF burden. |  |
| Primary endpoint | Composite of IS, TIA and SE. | Composite of all-cause mortality, incidence of clinical AF, thromboembolic events (IS, TIA, systemic thromboembolism). | CV mortality. | Composite of ischemic stroke and TIA. | Composite of progression to clinical AF, IS, MI, HF hospitalization and cardiac death. |  |
| Secondary endpoints |  | Incidence of clinical AF, incidence of thromboembolic events. | All-cause mortality, stroke, non-CV mortality. |  | Each component of the primary outcome. |  |
| Sponsor | China Scholarship Council | NA | NA | NA | None | NA |

| Study Name | **Miyakawa 2019** | **Nakano 2019** | **Chu 2020** | **Martin 2015**  **(IMPACT)** | **Bertini 2010** |
| --- | --- | --- | --- | --- | --- |
| General characteristics | Retrospective, single centre | Retrospective, single centre | Retrospective, single centre | Randomized, multicentre clinical trial | Prospective, multicentre |
| Patient population | Patients with CIED. | Patients with CIED and no history of AF or anticoagulation therapy. | Patients with CIED. | Patients with ICD or CRT-D and CHADS2 >=1. | Patients with CIED and HF. |
| Number of patients | 856 (644 with no history of AF) | 348 | 152 | 2718 | 393 |
| Number of patients with AHRE | 125 (50 with no history of AF) | 75 (55> 30”) | 56 | 571 | 84 |
| Year of publication | 2019 | 2019 | 2020 | 2015 | 2010 |
| OAC use at baseline |  | 0% | 0% | 0% | 53% |
| ICM device | Dual chamber PM, ICD, CRT | Dual chamber PM, ICD | Dual chamber PM | ICD, CRT-D | ICD, CRT |
| Follow-up monitoring strategy | Routine clinical follow-up | Clinical visits and device interrogation.every 6-month interval | Clinical visits and device interrogation at 3, 6 months after implantation and then annually. | Regular clinical follow-up +/- home monitoring | Routine clinical follow-up |
| AHRE/subclinical AF definition | Any AT with an atrial rate ≥ 175 lasting for at least 5 minutes (required EGM confirmation) | Any AT with an atrial rate > 175, 190, and 200 beats/ min according to the Medtronic, Abbott, and Biotronik devices, respectively, lasting for at least 30 s (required EGM confirmation) | Any AT with an atrial rate > 160 bpm lasting for at least 6 minutes. | Any AT with an atrial rate ≥ 200 bpm lasting for at least 6 minutes. | Any AT with an atrial rate > 180 lasting for at least 10 minutes in patients with CRT/Dual-chamber ICD. In single- chamber devices, with device-based diagnostics. |
| Major events definition | Ischemic stroke, TIA, systemic embolism | Ischemic stroke confirmed by imaging | IS, TIA and SE | Ischemic stroke confirmed by imaging Systemic thromboembolism confirmed by angiography. | Ischemic stroke |
| Average FU | 48.2 months | 65 months | 67 months | 23 months | 16 months |
| Major inclusion criteria | - Implantation of PM, ICD, or CRT | - Implantation of PM or ICD | - Biotronik dual-chamber PM implantation | - Patients with ICD or CRT-D. - CHADS2 risk score ≥1 - Ability to tolerate anticoagulation. | - Patients with mild to severe HF scheduled for ICD implantation for primary or secondary prevention according to the current AHA/ACC/ESC guidelines. |
| Major exclusion criteria | - Single-chamber CIED. - Less than 6 months of follow-up. | - History of AF - Anticoagulation therapy. - Single chamber CIED | - Age < 18. - History of thromboembolism. - Anticoagulation therapy. | - Permanent AF - Contraindications to anticoagulation. | - Absence of normal sinus rhythm during echocardiographic examination. - Acoustic window with poor image quality. |
| Primary endpoint | Composite of IS, TIA, systemic embolism or all cause death | Ischemic stroke | Composite of IS, TIA or SE. | Composite of stroke, SE, and major  bleeding. | Identifying clinical and/or echocardiographic predictors of AF occurrence, including LA volumes, LA function, and PA-TDI duration, in patients with heart failure |
| Secondary endpoints |  |  |  | Rate of all-cause mortality, IS and hemorrhagic stroke, fatal or disabling and non-disabling stroke, major bleeding events, AF/AFL burden, cardioembolic and non-cardioembolic stroke, change in QoL score. |  |
| Sponsor | None | None | Youth clinical research project of Peking University First Hospital. | Biotronik Inc. | NA |

ACC, American College of Cardiology; AF, atrial fibrillation; AFL, atrial flutter; AHA, American Heart Association; AHRE, atrial high rate episode; AT, atrial tachyarrhythmia; AV, atrioventricular; BB, bundle branch; CHF, chronic heart failure; CIED, cardiac implantable electronic device; CRT-P/D, cardiac resynchronization therapy-pacemaker/defibrillator; CV, cardiovascular; DAPT, dual antiplatelet therapy; EGM, electrogram; EHR, electronic health record; ESC, European Society of Cardiology; HF, heart failure; ICD, implantable cardioverter defibrillator; IS, ischemic stroke; LA, left atrial; MI, myocardial infarction; NA: not available/applicable; PAE: peripheral artery embolism; PAF, paroxysmal atrial fibrillation; PA-TDI duration, time-interval from the beginning of the electrocardiogram P wave and the peak of A′lateral wave at tissue Doppler images; PerAF, persistent atrial fibrillation; PM, pacemaker; QoL, quality of life; SCAF, subclinical atrial fibrillation; SE, systemic embolism; SND, sinus node disease; TIA, transient ischemic attack; VKA, vitamin K antagonist.

**Table S3.** TP, TN, FP and FN counts for the included studies, at each evaluated cut-off (longest AHRE duration)

| **Study** | **Year** | **Cutoff_min** | **TP** | **TN** | **FP** | **FN** |
| --- | --- | --- | --- | --- | --- | --- |
| Kim | 2016 | 5 | 7 | 752 | 115 | 6 |
| Kaplan | 2019 | 6 | 177 | 16338 | 4776 | 477 |
| Kaplan | 2019 | 1410 | 67 | 19609 | 1505 | 587 |
| VanGelder | 2017 | 6 | 16 | 1792 | 528 | 19 |
| VanGelder | 2017 | 360 | 9 | 2095 | 225 | 26 |
| VanGelder | 2017 | 1440 | 7 | 2198 | 122 | 28 |
| Caldwell | 2009 | 0,5 | 1 | 73 | 26 | 1 |
| Kawakami | 2017 | 6 | 4 | 160 | 92 | 5 |
| Miyazawa | 2019 | 5 | 3 | 571 | 47 | 23 |
| Nakano | 2019 | 0,5 | 13 | 277 | 48 | 10 |
| Nakano | 2019 | 1 | 9 | 283 | 42 | 14 |
| Nakano | 2019 | 6 | 5 | 295 | 30 | 18 |
| Nakano | 2019 | 10 | 4 | 301 | 24 | 19 |
| Nakano | 2019 | 30 | 3 | 310 | 15 | 20 |
| Nakano | 2019 | 60 | 2 | 315 | 10 | 21 |
| Nakano | 2019 | 1440 | 0 | 323 | 2 | 23 |
| Healey | 2012 | 6 | 11 | 2279 | 250 | 40 |
| Li | 2019 | 5 | 15 | 401 | 160 | 18 |
| Petrac | 2012 | 5 | 9 | 195 | 67 | 3 |
| Witt | 2015 | 6 | 11 | 296 | 68 | 19 |
| Gonzalez | 2014 | 5 | 3 | 183 | 36 | 2 |
| Lu | 2021 | 0,5 | 19 | 193 | 143 | 0 |
| Lu | 2021 | 1 | 19 | 210 | 126 | 0 |
| Lu | 2021 | 2 | 17 | 228 | 108 | 2 |
| Lu | 2021 | 5 | 14 | 243 | 93 | 5 |
| Lu | 2021 | 360 | 6 | 287 | 49 | 13 |
| Lu | 2021 | 1440 | 5 | 304 | 32 | 14 |
| O'Neill | 2018 | 6 | 5 | 80 | 49 | 10 |
| Park | 2021 | 6 | 5 | 151 | 339 | 1 |
| Sandgren | 2018 | 5 | 13 | 266 | 112 | 20 |
| Nishinarita | 2019 | 5 | 2 | 70 | 32 | 0 |
| Bertini | 2010 | 10 | 1 | 305 | 83 | 4 |
| Chu | 2020 | 6 | 5 | 91 | 51 | 5 |
| Chu | 2020 | 60 | 3 | 107 | 35 | 7 |
| Chu | 2020 | 360 | 1 | 114 | 28 | 9 |
| Chu | 2020 | 1440 | 0 | 127 | 15 | 10 |

**Table S4.** TP, TN, FP and FN counts for the included studies, at each evaluated cut-off (cumulative AHRE burden)

| **Study** | **Year** | **Cutoff_min** | **TP** | **TN** | **FP** | **FN** |
| --- | --- | --- | --- | --- | --- | --- |
| Martin | 2015 | 6 | 20 | 2098 | 551 | 49 |
| Martin | 2015 | 330 | 15 | 2360 | 289 | 54 |
| Amara | 2017 | 360 | 5 | 440 | 144 | 6 |
| Boriani | 2014 | 5 | 58 | 5688 | 4229 | 41 |
| Boriani | 2014 | 60 | 50 | 6612 | 3305 | 49 |
| Boriani | 2014 | 360 | 36 | 7412 | 2505 | 63 |
| Boriani | 2014 | 720 | 31 | 7872 | 2045 | 68 |
| Boriani | 2014 | 1380 | 25 | 8386 | 1531 | 74 |
| Shanmugan | 2012 | 14 | 5 | 256 | 121 | 0 |

**Table S5.** Number of studies for each temporal cut-off (primary analysis, longest AHRE episode)

| **Time cut-off (min)** | **Number of studies** |
| --- | --- |
| 0.5 | 3 |
| 1 | 2 |
| 2 | 1 |
| 5 | 8 |
| 6 | 9 |
| 10 | 2 |
| 30 | 1 |
| 60 | 2 |
| 360 | 3 |
| 1410 | 1 |
| 1440 | 4 |

**Table S6.** Number of studies for each temporal cut-off (primary analysis, cumulative AHRE burden)

| **Time cut-off (min)** | **Number of studies** |
| --- | --- |
| 5 | 1 |
| 6 | 1 |
| 14 | 1 |
| 60 | 1 |
| 330 | 1 |
| 360 | 2 |
| 720 | 1 |
| 1380 | 1 |

**Supplementary figures**

**Figure S1.** Meta-analysis of diagnostic test accuracy studies with multiple time cut-offs assessing the differential performances of various longest AHRE duration thresholds in predicting thromboembolic events (only studies including patients without prior history of AF). A) Youden index as a function of test thresholds; B) Study-specific ROC curves; C) Meta-analytic SROC curve; D) Meta-analytic sensitivity/specificity as a function of different test thresholds.

**Figure S2.** Meta-analytic forest plot (random effects model) reporting the prevalence of thromboembolic events in patients with CIED analyzing longest AHRE duration (only studies including patients without prior history of AF).

**Figure S3**. Meta-analysis of diagnostic test accuracy studies with multiple time cut-offs assessing the differential performances of various longest AHRE duration thresholds in predicting thromboembolic events (high quality studies only). A) Youden index as a function of test thresholds; B) Study-specific ROC curves; C) Meta-analytic SROC curve; D) Meta-analytic sensitivity/specificity as a function of different test thresholds.

**Figure S4.** Meta-analytic forest plot (random effects model) reporting the prevalence of thromboembolic events in patients with CIED analyzing longest AHRE duration (high quality studies only).

**Supplementary References**

1. Pothineni NVK, Amankwah N, Santangeli P, Schaller RD, Supple GE, Deo R, *et al.* Continuous rhythm monitoring-guided anticoagulation after atrial fibrillation ablation. *J Cardiovasc Electrophysiol* 2021;**32**:345–53.

2. Wilton SB, Exner D V., Wyse DG, Yetisir E, Wells G, Tang ASL, *et al.* Frequency and Outcomes of Postrandomization Atrial Tachyarrhythmias in the Resynchronization/Defibrillation in Ambulatory Heart Failure Trial. *Circ Arrhythmia Electrophysiol* 2016;**9**:1–9.

3. Bertini M, Borleffs CJW, Delgado V, Ng ACT, Piers SRD, Shanks M, *et al.* Prediction of atrial fibrillation in patients with an implantable cardioverter-defibrillator and heart failure. *Eur J Heart Fail* 2010;**12**:1101–10.

4. Nishinarita R, Niwano S, Fukaya H, Oikawa J, Nabeta T, Matsuura G, *et al.* Burden of implanted-device-detected atrial high-rate episode is associated with future heart failure events: - Clinical significance of asymptomatic atrial fibrillation in patients with implantable cardiac electronic devices. *Circ J* 2019;**83**:736–42.

5. Petrač D, Radeljić V, Delić-Brkljačić D, Manola Š, Cindrić-Bogdan G, Pavlović N. Persistent atrial fibrillation is associated with a poor prognosis in patients with atrioventricular block and dual-chamber pacemaker. *PACE - Pacing Clin Electrophysiol* 2012;**35**:695–702.

6. Kim BS, Chun KJ, Hwang JK, Park SJ, Park KM, Kim JS, *et al.* Predictors and long-term clinical outcomes of newly developed atrial fibrillation in patients with cardiac implantable electronic devices. *Med (United States)* 2016;**95**.

7. Kaplan RM, Koehler J, Ziegler PD, Sarkar S, Zweibel S, Passman RS. Stroke risk as a function of atrial fibrillation duration and CHA2DS2-VASc score. *Circulation* 2019;**140**:1639–46.

8. Gelder IC Van, Healey JS, Crijns HJGM, Wang J, Hohnloser SH, Gold MR, *et al.* Duration of device-detected subclinical atrial fibrillation and occurrence of stroke in ASSERT. *Eur Heart J* 2017;**38**:1339–44.

9. Amara W, Montagnier C, Cheggour S, Boursier M, Gully C, Barnay C, *et al.* Early Detection and Treatment of Atrial Arrhythmias Alleviates the Arrhythmic Burden in Paced Patients: The SETAM Study. *PACE - Pacing Clin Electrophysiol* 2017;**40**:527–36.

10. Boriani G, Glotzer T V., Santini M, West TM, Melis M De, Sepsi M, *et al.* Device-detected atrial fibrillation and risk for stroke: An analysis of >10 000 patients from the SOS AF project (Stroke preventiOn Strategies based on Atrial Fibrillation information from implanted devices). *Eur Heart J* 2014;**35**:508–16.

11. Caldwell JC, Contractor H, Petkar S, Ali R, Clarke B, Garratt CJ, *et al.* Atrial fibrillation is under-recognized in chronic heart failure: Insights from a heart failure cohort treated with cardiac resynchronization therapy. *Europace* 2009;**11**:1295–300.

12. Shanmugam N, Boerdlein A, Proff J, Ong P, Valencia O, Maier SKG, *et al.* Detection of atrial high-rate events by continuous Home Monitoring: Clinical significance in the heart failurecardiac resynchronization therapy population. *Europace* 2012;**14**:230–7.

13. Kawakami H, Nagai T, Saito M, Inaba S, Seike F, Nishimura K, *et al.* Clinical significance of atrial high-rate episodes for thromboembolic events in Japanese population. *Heart Asia* 2017;**9**:e010954.

14. Miyazawa K, Pastori D, Martin DT, Choucair WK, Halperin JL, Lip GYH. Characteristics of patients with atrial high rate episodes detected by implanted defibrillator and resynchronization devices. *Europace* 2022;**24**:375–83.

15. Nakano M, Kondo Y, Nakano M, Kajiyama T, Hayashi T, Ito R, *et al.* Impact of atrial high-rate episodes on the risk of future stroke. *J Cardiol* Japanese College of Cardiology; 2019;**74**:144–9.

16. Healey JS, Connolly SJ, Gold MR, Israel CW, Gelder IC Van, Capucci A, *et al.* Subclinical Atrial Fibrillation and the Risk of Stroke. *N Engl J Med* 2012;**366**:120–9.

17. Li YG, Miyazawa K, Pastori D, Szekely O, Shahid F, Lip GYH. Atrial high-rate episodes and thromboembolism in patients without atrial fibrillation: The West Birmingham Atrial Fibrillation Project. *Int J Cardiol* Elsevier B.V.; 2019;**292**:126–30.

18. Witt CT, Kronborg MB, Nohr EA, Mortensen PT, Gerdes C, Nielsen JC. Early detection of atrial high rate episodes predicts atrial fibrillation and thromboembolic events in patients with cardiac resynchronization therapy. *Hear Rhythm* Elsevier; 2015;**12**:2368–75.

19. Gonzalez M, Keating RJ, Markowitz SM, Liu CF, Thomas G, Ip JE, *et al.* Newly detected atrial high rate episodes predict long-term mortality outcomes in patients with permanent pacemakers. *Hear Rhythm* Elsevier; 2014;**11**:2214–21.

20. Lu W Da, Chen JY. The optimal cutoff of atrial high-rate episodes for neurological events in patients with dual chamber permanent pacemakers. *Clin Cardiol* 2021;**44**:871–9.

21. O’Neill J, Jegodzinski L, Tayebjee MH. Incidence of subclinical atrial fibrillation in a South Asian population. *PACE - Pacing Clin Electrophysiol* 2018;**41**:1600–5.

22. Park YJ, Kim JS, Park KM, On YK, Park SJ. Subclinical Atrial Fibrillation Burden and Adverse Clinical Outcomes in Patients with Permanent Pacemakers. *Stroke* 2021;1299–308.

23. Sandgren E, Rorsman C, Edvardsson N, Engdahl J. Stroke incidence and anticoagulation treatment in patients with pacemaker-detected silent atrial fibrillation. *PLoS One* 2018;**13**:1–12.

24. Chu SY, Jiang J, Wang YL, Sheng QH, Zhou J, Ding YS. Pacemaker-detected atrial fibrillation burden and risk of ischemic stroke or thromboembolic events—A cohort study. *Hear Lung* Elsevier Inc.; 2020;**49**:66–72.

25. Martin DT, Bersohn MM, L.waldo A, Wathen MS, Choucair WK, Lip GYH, *et al.* Randomized trial of atrial arrhythmia monitoring to guide anticoagulation in patients with implanted defibrillator and cardiac resynchronization devices. *Eur Heart J* 2015;**36**:1660–8.
